# New Insights on the Maternal Diet Induced-Hypertension: Potential Role of the Phenotypic Plasticity and Sympathetic-Respiratory Overactivity

**DOI:** 10.3389/fphys.2015.00345

**Published:** 2015-11-24

**Authors:** João H. Costa-Silva, José L. de Brito-Alves, Monique Assis de V. Barros, Viviane Oliveira Nogueira, Kássya M. Paulino-Silva, Allan de Oliveira-Lira, Isabele G. Nobre, Jéssica Fragoso, Carol G. Leandro

**Affiliations:** Department of Physical Education and Sport Science, Academic Center of Vitoria, Federal University of PernambucoVitória de Santo Antão, Brazil

**Keywords:** hypertension, developmental plasticity, perinatal nutrition, respiratory control, protein restriction

## Abstract

Systemic arterial hypertension (SAH) is an important risk factor for cardiovascular disease and affects worldwide population. Current environment including life style coupled with genetic programming have been attributed to the rising incidence of hypertension. Besides, environmental conditions during perinatal development such as maternal malnutrition can program changes in the integration among renal, neural, and endocrine system leading to hypertension. This phenomenon is termed phenotypic plasticity and refers to the adjustment of a phenotype in response to environmental stimuli without genetic change, following a novel or unusual input during development. Human and animal studies indicate that fetal exposure to an adverse maternal environment may alter the renal morphology and physiology that contribute to the development of hypertension. Recently, it has been shown that the maternal protein restriction alter the central control of SAH by a mechanism that include respiratory dysfunction and enhanced sympathetic-respiratory coupling at early life, which may contribute to adult hypertension. This review will address the new insights on the maternal diet induced-hypertension that include the potential role of the phenotypic plasticity, specifically the perinatal protein malnutrition, and sympathetic-respiratory overactivity.

## Introduction

Hypertension is a highly prevalent and significant risk factor for the development of metabolic disease, including coronary heart disease (CHD), stroke, heart failure, aortic, and peripheral arterial disease (Landsberg et al., 2013). The etiology of hypertension includes a complex phenotype that arises from numerous genetic, environmental, behavioral, ethnic, and even social origins (Landsberg et al., 2013). However, it has been observed that the perinatal nutritional milieu during “sensitive” periods of development or in infant affects the normal growth/developing and this may be associated with adult disease (Lucas, 1998; Victora et al., 2008; Wells, 2012). This phenomenon can be understood in the context of phenotypic plasticity. Phenotypic plasticity refers to the ability of an organism to react to an internal and external environmental input with a change in the form, state, movement, or rate of activity without genetic changes (West-Eberhard, 2005).

The association between Systemic arterial hypertension (SAH) and nutritional factors has been studied by a large number of epidemiological and clinical studies (Ashton, 2000; Hemachandra et al., 2006; Antony and Laxmaiah, 2008; Conde and Monteiro, 2014; Parra et al., 2015). Indeed, perinatal malnutrition is associated with the risk of developing cardiovascular disease and co-morbidities in later life including hypertension, metabolic syndrome and diabetes, (Nuyt, 2008; Nuyt and Alexander, 2009). In humans, studies have provided support for the positive association between low birth weight and increased incidence of hypertension (Ravelli et al., 1976; Hales et al., 1991; Sawaya and Roberts, 2003; Sawaya et al., 2004).

It is well established that perinatal malnutrition environmental stimuli can contribute to the programming of subsequent risks of hypertension by mechanisms that include reduced nephron morphology and function, reduced glomerular filtration rate, and dysfunction on the renin-angiotensin-aldosterone system (Nuyt and Alexander, 2009). Recently, studies have highlighted the contribution of the sympathetic-respiratory dysfunctions on the development of the maternal diet induced-hypertension (de Brito Alves et al., 2015). Protein-restricted rats during gestation and lactation showed respiratory dysfunction, which was associated with sympathetic overactivity and enhanced carotid bodies (CB) sensitivity to hypoxia (de Brito Alves et al., 2015; Nanduri and Prabhakar, 2015; Prabhakar et al., 2015). The underlying mechanism may be associated with high levels of hypoxic inducible factor (HIF-1α) in CB peripheral chemoreceptor (Ito et al., 2011, 2012; de Brito Alves et al., 2015). Thus, the aim of this review was to address the new insights about maternal diet induced-hypertension and the concept that perinatal malnutrition may affect the ventilatory and cardiovascular control.

## New insights on the perinatal origin of hypertension: The role of phenotypic plasticity

**Figure 1:**
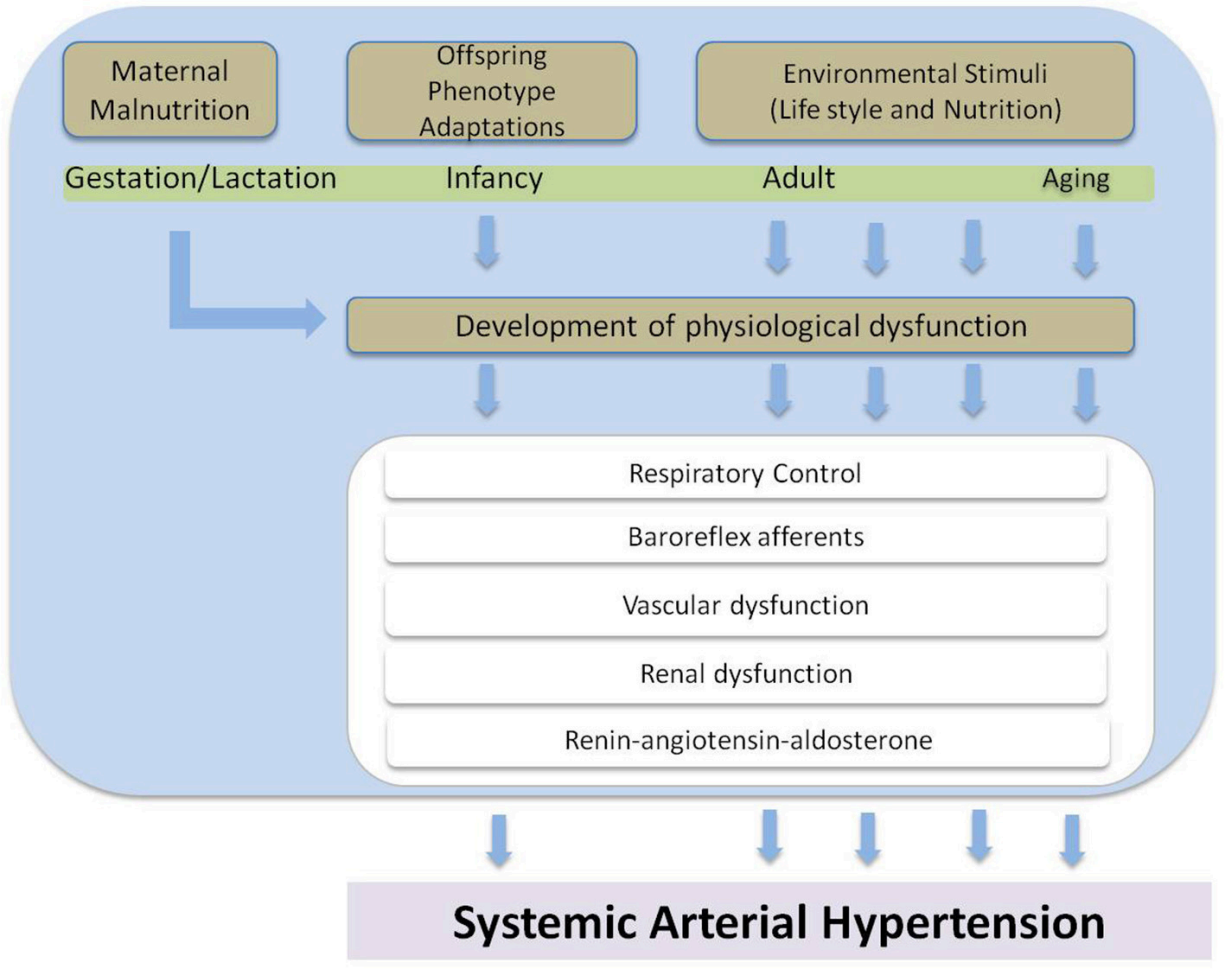
**Overview about the environmental influence during development on early appearance of systemic arterial hypertension**.

One of the best-known attempts to understand the association between early nutrition and SAH is the “thrifty phenotype hypothesis” proposed by Hales and Barker in 1992. This hypothesis is extensively used to consider cardiovascular disease as a “programmed” effect of nutritional restriction during early phases of growth and development, followed by a recovery of the diet during lifespan (Hales and Barker, 1992). This phenomenon can be understood in the context of the phenotypic plasticity (Barker et al., 2005; West-Eberhard, 2005; Labayen et al., 2006; Andersen et al., 2009; Biosca et al., 2011). Phenotypic plasticity is defined as the ability of an organism to react to an environmental stimuli with a adaptive mutual adjustment, without genetic change, among variable aspects of the phenotype, following a novel or unusual input during development (West-Eberhard, 2005). Epigenetic alterations as DNA methylation, histone acetylation and microRNA expression are the molecular basis of the phenotypic plasticity (Wells, 2011). Maternal low-protein diet model during gestation and/or lactation is one of the most extensively studied animal models of phenotypic plasticity (Ozanne and Hales, 2004; Costa-Silva et al., 2009; Falcão-Tebas et al., 2012; Fidalgo et al., 2013; de Brito Alves et al., 2014). An overview about the environmental influence during development on early appearance of SAH is shown in Figure 1. Feeding a low-protein diet (8% casein) during gestation followed by the consumption of a normocaloric diet throughout lactation is associated with growth restriction, asymmetric reduction in organ growth, elevated systolic blood pressure, and increased fasting plasma insulin concentrations (Ozanne and Hales, 2004; Fidalgo et al., 2013; de Brito Alves et al., 2014). Recently, it was demonstrated that adult animals subjected to maternal protein restriction presented mainly an increase in the cardiovascular sympathetic tone and increased low frequency (LF) bands of the SAH, suggesting autonomic misbalance, and sympathetic predominance on the cardiovascular system of these animals (Barros et al., 2015).

It is known that the rhythmicity of the sympathetic nervous system can modulate the arterial pressure (AP) and the heart rate at regular frequencies (Tseng et al., 2009). These rhythmic fluctuations in the cardiovascular variables suggest a measurement of cardiovascular autonomic balance (Japundzic-Zigon, 1998). Accordingly, the LF oscillations of the systolic arterial pressure (SAP) are typically enhanced during states of sympathetic activation (Julien, 2006) and are increased in the offspring from dams subjected to protein restriction during perinatal period and may contribute to the development of arterial hypertension in this experimental model (de Brito Alves et al., 2015).

Although the relationship between maternal protein restriction and sympathetic overactivity have been suggested (Johansson et al., 2007; Franco et al., 2008; Barros et al., 2015), less is known about the physiological dysfunctions responsible for producing these effects. In this context, it is described that a baroreflex dysfunction could lead to a sympathetic overactivity and subsequent development of hypertension (Souza et al., 2001; Heusser et al., 2010; Tsyrlin et al., 2013). However, the hypothesis that maternal protein restriction leads to baroreflex dysfunction has not been proved yet.

Nowadays, it is well accepted that perinatal protein malnutrition raise risks of hypertension by mechanisms that include reduced nephron morphology and function, and dysfunction on the renin-angiotensin system (Chen et al., 2010; Siddique et al., 2014). However, other hypotheses have been highlighted considering the role of sympathetic overactivity and the development of hypertension in organisms that suffered perinatal malnutrition. In different models of hypertension, it has been suggested that changes in the generation or modulation of respiratory function can contribute to the development of arterial hypertension (Simms et al., 2009, 2010; Costa-Silva et al., 2012; Moraes et al., 2014). Indeed, respiratory neurons located into the brainstem may modulate the sympathetic nervous system and the levels of arterial pressure by central pathways (Costa-Silva et al., 2009, 2010, 2012; Moraes et al., 2014). These neurons receive strong influences from peripheral respiratory chemoreceptors, located into CB at the aortic and carotid arteries. Activation of these chemoreceptors produces a powerful activation of the cardiorespiratory neuronal network and enhances the sympathetic outflow and respiratory drive, which are essential to cardiovascular and ventilatory stability and for providing a correct O_2_ delivery to cells (Costa-Silva et al., 2010, 2012; Moraes et al., 2014). Thus, it has been suggested that CB dysfunction induced by phenotypic plasticity at the early life can lead to autonomic and ventilatory disorders (Nanduri and Prabhakar, 2015; Prabhakar et al., 2015).

**Figure 2:**
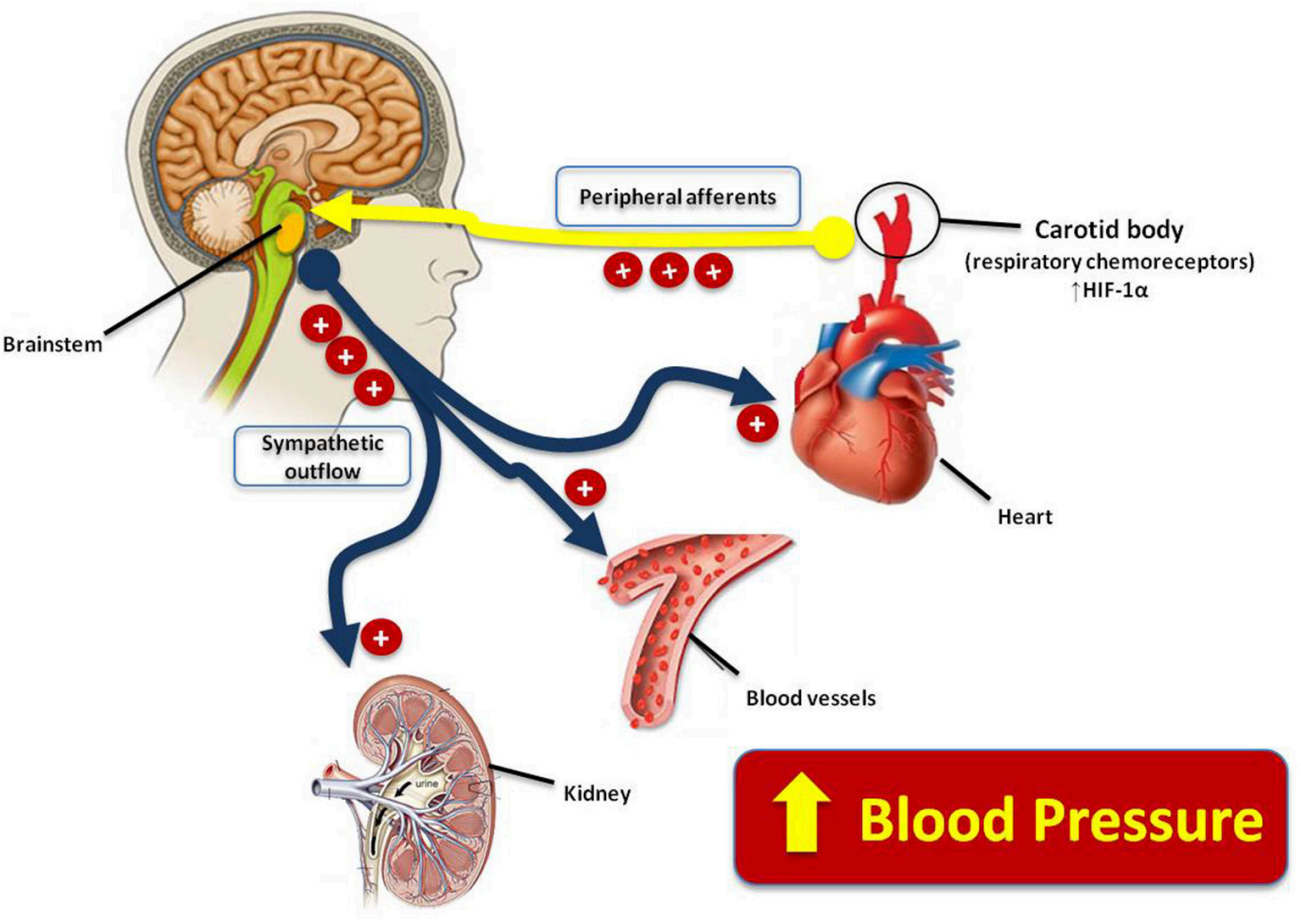
**Schematic drawing showing the new insights on the maternal diet induced-hypertension, and the influence of the augmented afferent inputs from the carotid body (peripheral respiratory chemoreceptors) to brainstem and enhanced sympathetic outflow to the kidney, heart and blood vessels, which may lead to increased blood pressure in the adult offspring subjected to maternal protein restriction**.

Recently, experimental studies showed that maternal protein restriction during pregnancy and lactation leads to relevant short-term effects on the CB sensitivity and respiratory control of the offspring (de Brito Alves et al., 2014, 2015). Maternal protein restriction is able to induce high phrenic burst frequency and amplitude, leading to increased baseline respiratory frequency (up to 28%) and ventilation (up to 40%) (de Brito Alves et al., 2014, 2015). Further, studies *in situ* also observed that these respiratory dysfunctions are associated with enhanced baseline sympathetic activity and amplified ventilatory and sympathetic responses to peripheral chemoreflex activation prior to the establishment of hypertension, and high ventilatory responses to hypoxia (de Brito Alves et al., 2015). Therefore, these data strongly support the hypothesis that protein-restricted rats have respiratory dysfunction, which was associated with sympathetic overactivity and enhanced CB sensitivity to hypoxia. Interestingly, this sympathetic-respiratory overactivity was associated with high levels of hypoxic inducible factor (HIF-1α) in CB peripheral chemoreceptor (de Brito Alves et al., 2015). Increased HIF-1α expression was previously observed in heart and brain from the protein-restricted animals (Ito et al., 2011, 2012) and support the notion that a high expression of this transcriptional factor (cellular response to hypoxia), is associated with enhanced sensory activity of the peripheral chemoreceptors, autonomic dysfunction, sympathetic overactivity, and increased risk of hypertension in the offspring subjected to maternal-protein restriction (Figure 2). However, the underlying mechanism involved in the HIF-1α up-regulation in protein-restricted rats is still unclear, but it is hypothesized that epigenetic mechanism produced by DNA methylation could be involved (Altobelli et al., 2013; Prabhakar, 2013; Nanduri and Prabhakar, 2015). Taken together, these studies reinforce the notion that the augmented afferent inputs from the CB (peripheral respiratory chemoreceptors) to brainstem and enhanced sympathetic outflow to the kidney, heart and blood vessels are highlighted as new insights on the maternal diet induced-hypertension, which may lead to increased blood pressure in the adult offspring subjected to maternal protein restriction.

## Conclusion

The etiology of the SAH is multifactorial involving genetic influences and the physiological integration of cardiovascular, renal, neural, and endocrine systems. Environmental stimuli are also strongly related to the high prevalence of SAH. Recently, it was recognize that perinatal malnutrition is related with the risk of developing metabolic syndrome and hypertension in adult life. The underlying mechanism can be explained in the context of phenotypic plasticity during development that includes adaptive change on the renal morphology and physiology with subsequent arterial hypertension. Moreover, maternal protein restriction may alter the central control of SAH by a mechanism that include enhanced sympathetic-respiratory activities and respiratory dysfunction at early life, which may contribute to adult hypertension. There are experimental evidences that respiratory dysfunction may be associated with both sympathetic overactivity and the high levels of HIF-1α in CB peripheral chemoreceptor.

## Author contributions

JC and CL drafted the work and revised critically for important intellectual content; wrote the paper; Final review of the manuscript. JB, VO, MA, KP, AO, IN, JF contributions to the conception of the work; Final review of the manuscript.

### Conflict of interest statement

The authors declare that the research was conducted in the absence of any commercial or financial relationships that could be construed as a potential conflict of interest.

## Abbreviations

SAH: systemic arterial hypertension
BP: Blood pressure
CB: carotid bodies
HF: High frequency
LF: low frequency
VLF: Very low frequency
AP: Arterial pressure
SAP: systemic arterial pressure.
